# Revealing the past of Ginah archaeological site by enhancing GPR images to understand ancient periods at Kharga Oasis, Egypt

**DOI:** 10.1038/s41598-025-10570-5

**Published:** 2025-07-21

**Authors:** Mohamed Osman Ebraheem, Hamza Ahmed Ibrahim, Mahmoud Mohamed Zalat

**Affiliations:** 1 Department of Geology, Faculty of Science, New Valley University, El-Kharga, 72511 New Valley Egypt; 2 Geology Department, Faculty of Science, Assiut University, Asyut, 71516 Egypt; 3 Cairo University, Cairo, Egypt

**Keywords:** Ground-penetrating radar, Georadar facies, Historical cultures, Ginah, Kharga Oasis, Egypt, Environmental sciences, Solid Earth sciences

## Abstract

Ground-penetrating radar (GPR) is a noninvasive near-surface geophysical method. This method is beneficial for imaging, characterization, and intrasite analysis of buried archaeological remains within culture sediments at Ginah archaeological site. The investigation of these targets has intrinsic value and has never been conducted at this site. In this study, GPR can be utilized to conduct a more focused survey on individual features and understand their structures, dimensions, and depths. The field survey on the studied area was conducted by SIR 4000 with 200 and 400-MHz antennae using RADAN 7 software. The processed GPR radargrams, depth slices, and 3D subvolumes are used to illustrate typical georadar facies associated with the stratigraphy and architectural elements of the buried archaeological remains. The facies analysis helps to identify the nature of cultural sediment, constructed materials, and the anticipated archaeological artifacts at various depths. These detected features are beneficial for presenting a compelling justification of nature, constituents, architectural patterns, and historical cultures. Also, this information is used to make guesses based on what is seen in the field and the archaeological history found in the ruins of Ginah, Al-Ghuieta, and Al-Zayyan fortresses along the Darb El-Arbine route. This information is essential to assume different successive ancient periods at the examined site, which can help specialists hasten their excavations.

## Introduction

Archaeologists can now employ geophysical surveying techniques to determine the optimum sites for excavation. The rapid development of geophysical technologies is beneficial for surveying sensitive buried archaeological sites remotely and nondestructively. This provides great facilities for detecting buried objects, saves time and effort, and protects fragile monuments^1–3^. GPR stands out among all applied geophysical methods, such as seismic refraction and electrical resistivity tomography, because it offers high-resolution, relative depth information in suitable soil conditions, particularly for near-surface features. Additionally, it can assist in investigations by contrasting the site’s natural soils with their archaeological components^4–7^. The localization of buried archaeological structures in subsurface soil can be detected because of the different contrasts in physical properties between the materials constituting the buried objects and the subsoil where they are preserved.

The archaeological potential of the Kharga Oasis represents one of the most interesting dimensions attracting the attention of specialists. Future excavations in the Oasis are expected to yield important ancient monuments from the Pharaonic to the Christian eras, which are currently largely unrecorded. The study of archaeological history in this region is important and has yielded significant results over the past decades^8–10^.

**Fig. 1:**
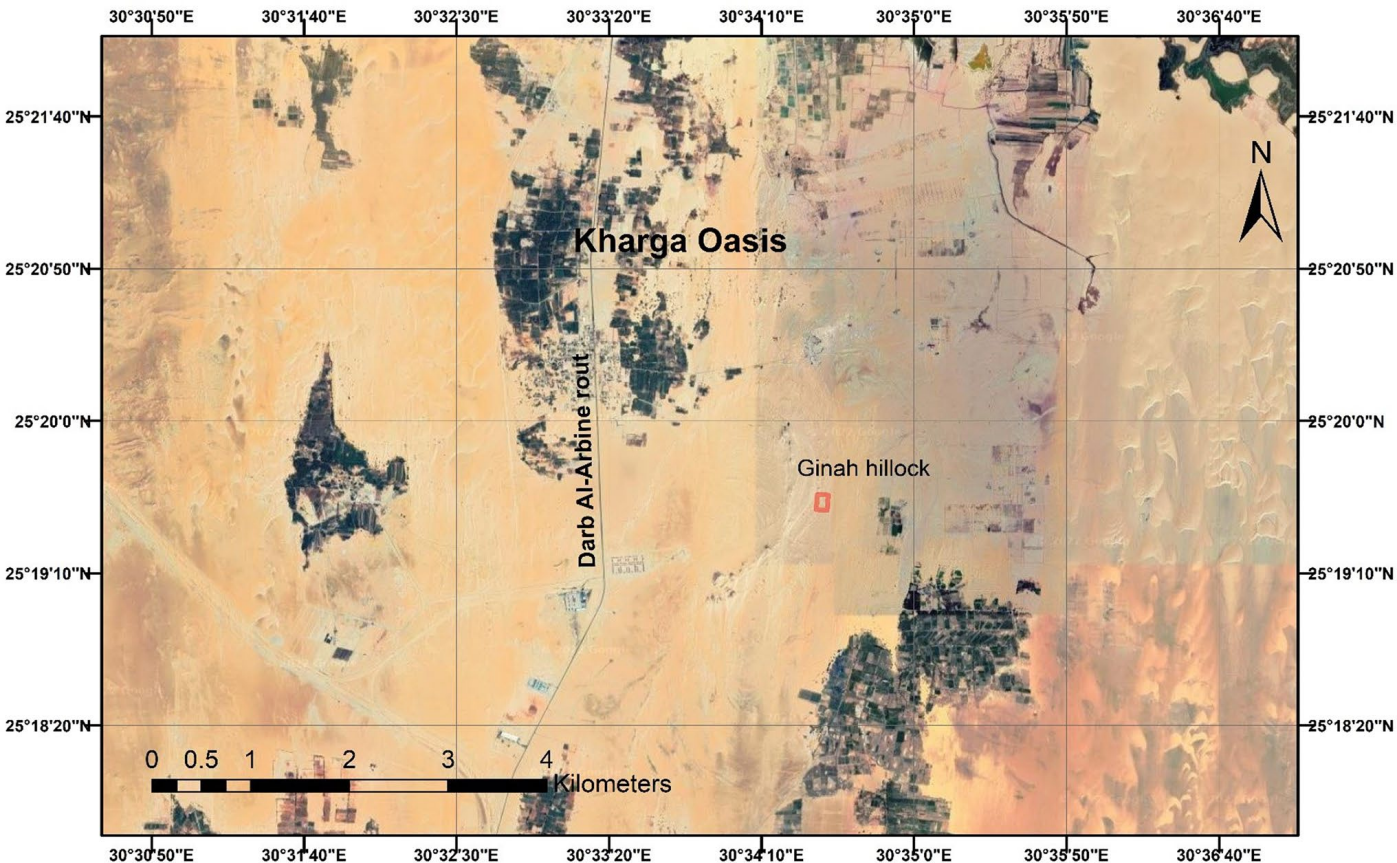
A satellite image showing; the location of Ginah Hillock and the Darb Al-Arbine route (was obtained from Wikipedia, source link; https://geology.com/world/egypt-satellite-image.shtml#google_vignette, then was modified by using Surfer software, Version 13.6, https://www.goldensoftware.com/surfer).

The Ginah Hillock was one of the fortress chains that controlled several caravan routes in the Roman and Ptolemaic periods (Fig. 1)^11^. It represents one of the most interesting and intensive activities across routes. Due to the lack of previously documented references and archaeological missions’ excavation activities, no one has historically been certain of the site’s purpose, origin, or nature. Were they first established as temples in the Pharaonic period, then turned into forts or fortresses in the Roman period, and finally turned into Christian habitations? This encourages the authors to reach a convincing explanation of the nature of the site. Therefore, the authors attempt to obtain a justified configuration by defining the following goals: (1) determine the corresponding relative dielectric permittivity and EM velocity of radar facies for buried culture sediments and archaeological objects; (2) characterize a variety of burials in different ancient periods; (3) compare architectural patterns and types of building materials for detected buried archaeological features for different periods; and (4) prepare assumptions of different archaeological burials in the area based on the remaining ruins on the surface and what has been inferred from the interpretation of the GPR data.

## Archaeological background

**Fig. 2:**
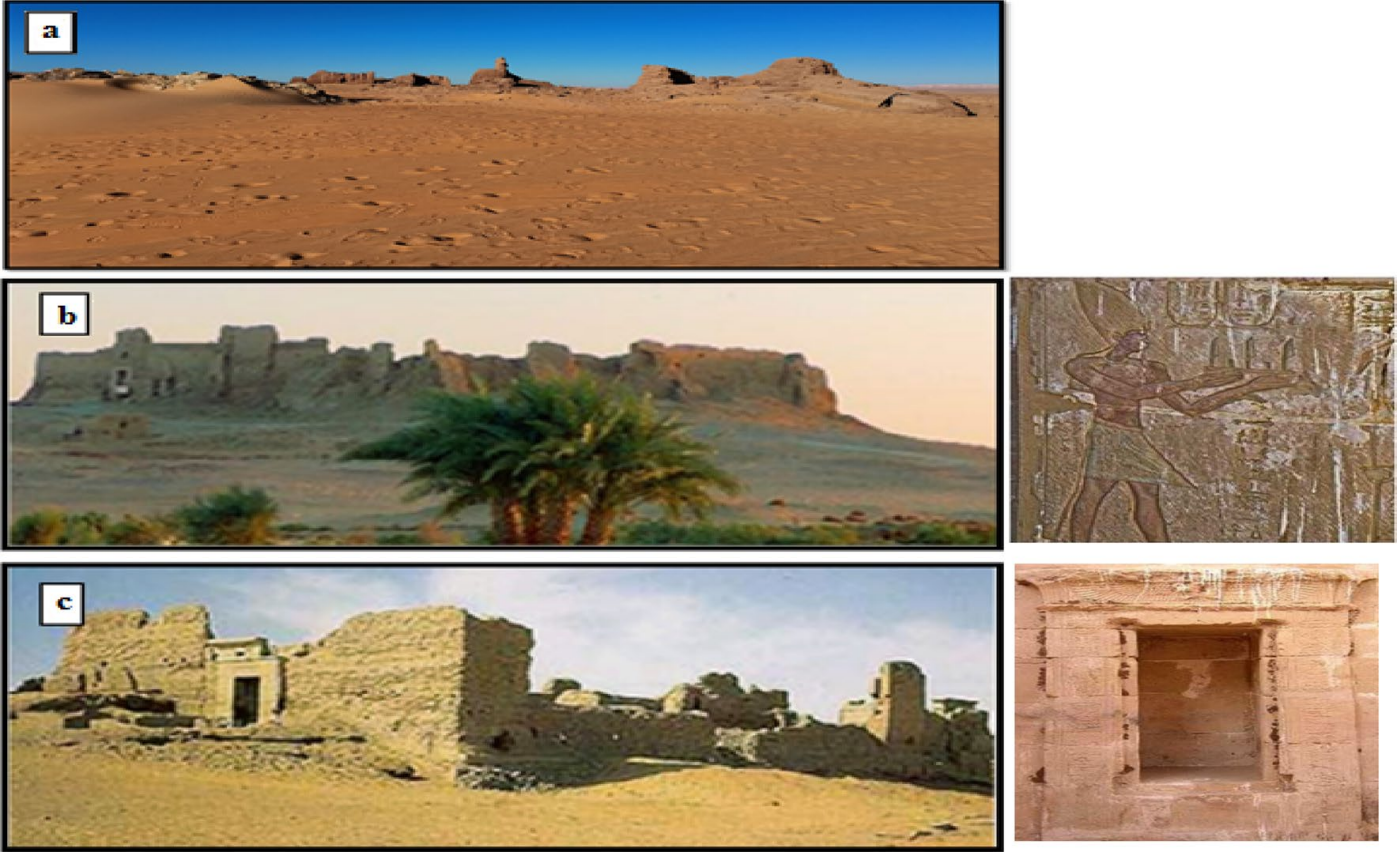
Panoramic view of; Ginah (**a**), El-Ghuieta (**b**), and El-Zayyan (**c**) fortresses. Adapted with permission from ref. ^36^ Copyright 2010 Elsevier Ltd.

The information suggests that human activity in the Kharga Oasis goes back around 40,000 years to the Upper Paleolithic period and extends into the Neolithic period in the fourth millennium^12–14^. Most of the archaeological sites in Kharga are related to the late Roman period (third–fifth century A.D.). The structures comprise a connected series of fortifications and fortified communities (such as Ginah, Al-Ghuieta, and Al-Zayyan) that serve military purposes (Fig. 2)^15^. They consist of an exceptional case of optimal use of the surrounding environment with their architectural design. No other area in Egypt has the same building system or anything that could be compared to it.

The archaeological site of Ginah probably follows an architectural style similar to that of most Roman fortresses. This site consists of mud bricks, especially the outside frame wall and the remaining water well^16^. Forts and their garrisons serve a variety of purposes. Additionally, they defended critical crossing points in the desert (Darb El-Arbine) or strategic points in the Western Desert from hostile populations (mostly bandits, Bedouin, Nobatae, and Blemmyes). Few forts in the Western Desert have been excavated. Forts, like the Ginah site, can occasionally be so destroyed that it is difficult to tell. The shapes of the forts varied in plan (rectilinear, oval, circular, and multiple). Small forts had fewer towers or none, while large ones had rectilinear, circular, or oval-shaped towers at the corners, on either side of the gates, and halfway along the walls. Stone was typically used for fort wall bases, whereas stone or mud brick was used for the superstructure. The walls were often tapered towards the top and frequently had a batter. Wells located in some areas store water channeled into their interior cisterns from nearby mountains. Many Egyptian Christians escaped to Kharga Oasis because of the unjust Romans, who persecuted the Copts of Egypt during the third and fourth centuries A.D. During this period, a few ancient temples and forts in the oasis were converted into churches and monasteries^17^.

**Fig. 3:**
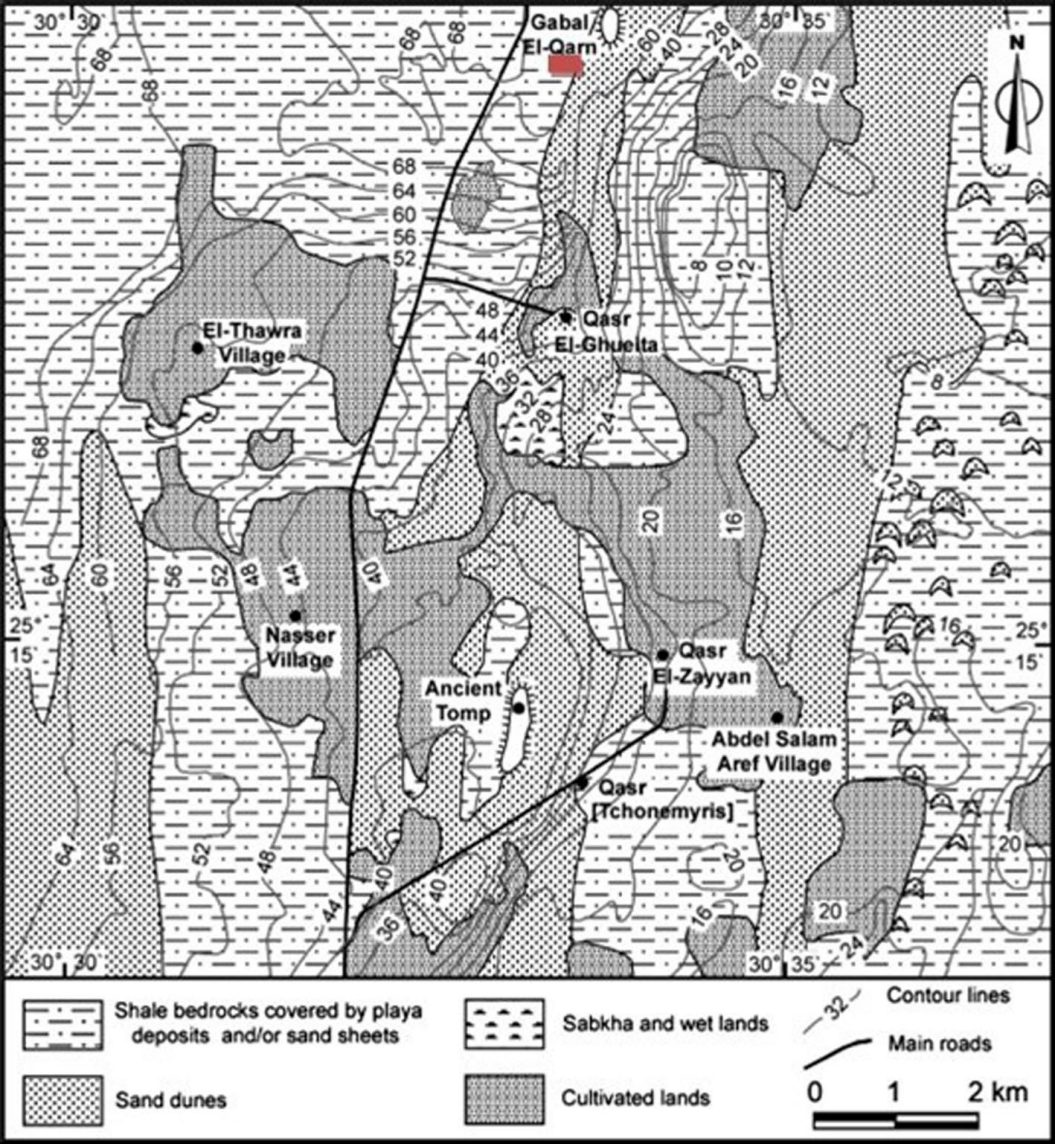
A simplified geological map of Kharga Oasis showing the location of the Ginah archaeological site. Adapted with permission from ref. ^36^ Copyright 2010 Elsevier Ltd.

**Fig. 4:**
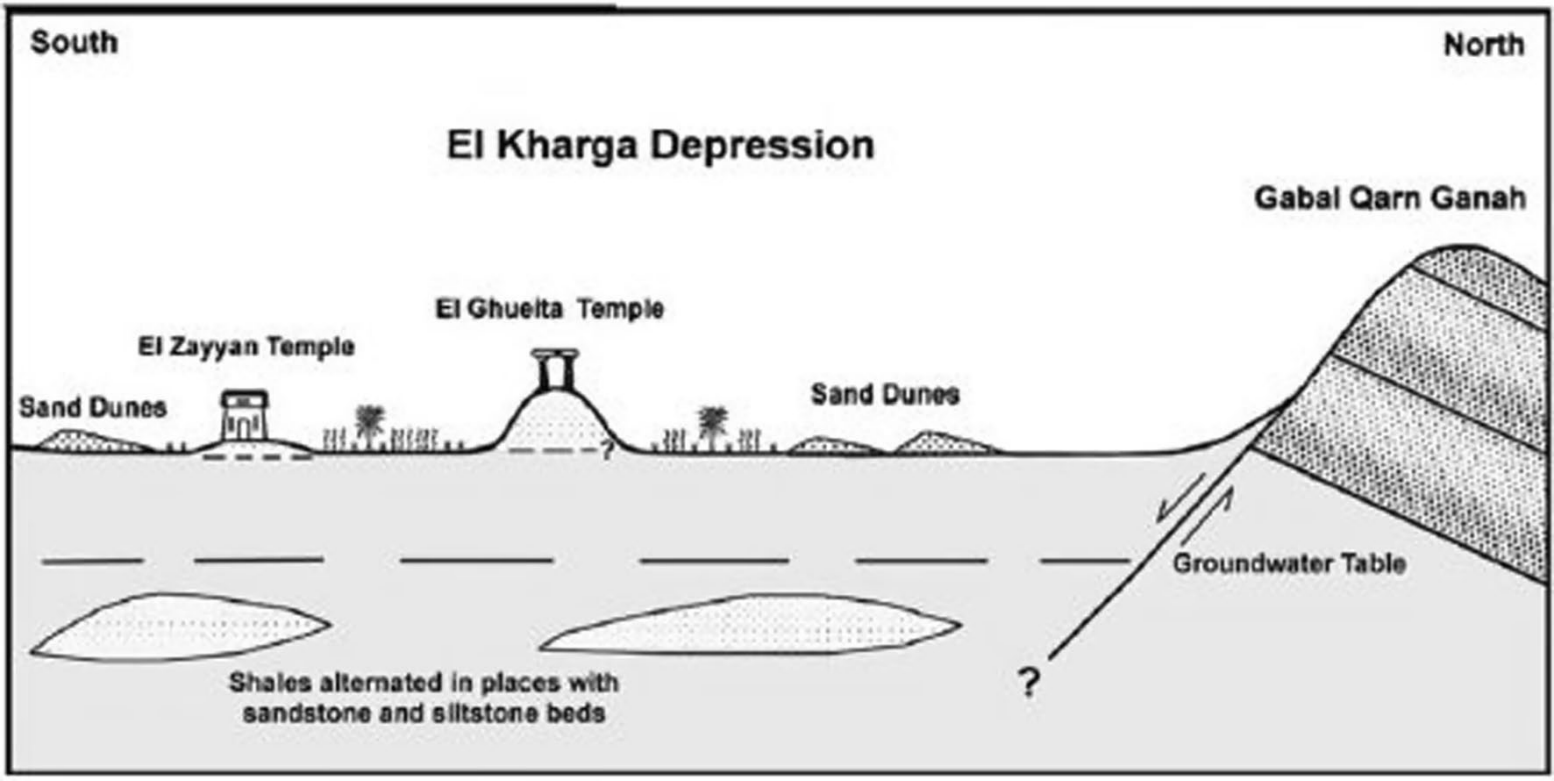
Sketch showing geologic and physiographic landscape characteristics of the area surrounding Ginah, El-Ghuieta, and El-Zayyan temples at Kharga Oasis. Adapted with permission from ref. ^36^ Copyright 2010 Elsevier Ltd.

The Oasis of Kharga consists of a varied natural environment dotted with archaeological remains from different periods. These various periods allow researchers and archaeologists to reevaluate the history and evolution of civilization at both regional and transnational scales. An oasis is considered an individual series of geomorphological and geological elements associated with the evolution of the local environment^18^. The structure of the Quarn Ginah dome is expressed topographically as conspicuous hillocks located north of the Al-Ghueita temple. It was started in the pre-Cretaceous period, as indicated by the fracture system in its sandstone layers belonging to the Taref Formation (Fig. 3)^19^. The archaeological sites (Ginah, Al-Ghuieta, and Al-Zayyan) are restricted to these hillocks because of their rocky terrain, which is suitable for the foundation of large structures (Fig. 4). These hillocks are composed of alternating sandstone, siltstone, and shale beds that constitute the bottom portion of the diverse rock unit of the Nubia Formation.

## GPR as an archaeogeophysical method

The field GPR survey depends mainly on transmitting an electromagnetic signal into the subsurface and the subsequent recording of the reflections. Two main factors (geometry and conductivity) influence the propagation of electromagnetic waves^20^. Due to the wave’s propagation into the medium, the EM wave velocity of GPR is a crucial parameter. Radar reflections can be used to detect subsurface contacts with a variety of petrophysical properties (Table 1)^21,22^. Other parameters, such as the shape and size of the target, frequency, intensity, polarization of the incident electromagnetic wave, and distance of the target from the antenna, must be considered when controlling the reflection capacity of the target.

**Table 1:** Typical dielectric constant, electrical conductivity, EM wave velocity, and attenuation values of common subsurface materials ^21,22^.

Material	Dielectric constant	Electrical conductivity (mSm^−1^)	EM wave velocity (m ns^−1^)	Attenuation (dB m^−1^)
Air	1	0	0.3	0
gravels	5	0.01	13	0.01
Fresh water	80	0.5	0.33	0.1
Limestone	4–8	0.5–2	0.12	0.4–1
Shale	5–15	1–100	0.09	1–100
Sandstone	5	0.01	0.13	0.01
Sand wet	20–30	0.1–1	0.06	0.03–0.3

GPR radargrams include many detailed reflections generated from subsurface features that are difficult to interpret^23^. Analysis of GPR data helps optimize the location and design of new excavations. Moreover, this information is widely used in excavation research, mainly aiming to discover potential buried remains over broad regions^24^, survey the foundations of current structures^25^, detect features or installations like ditches, walls, and roads^26,27^, find and visualize graves and burial sites^28^, and analyze ancient city locations^29^. More advanced techniques and software programs for GPR data acquisition and processing have been applied to archeological studies^30,31^. These methods enhance the imaging and characterization of buried structures by raising the overall resolution and the maximum penetration depth of buried archaeological structures^32^. In archaeological prospecting, GPR scans using 200–600 MHz antennas are advised. To attain a satisfactory resolution and lessen the attenuation impact that affects high frequencies, this decision was made^4^. On the other hand, because the physical contrasts characterizing submerged cultural history are frequently spatially irregular and not very voluminous, it is challenging to track continuous reflections together with cultural layers.

Depth-slices and 3D cubes generated via RADAN 7 software are excellent software techniques for further assessment and interpretation of field data. These methods create a 3D cube of the surveyed region by joining all of the separate data lines that were gathered using the designated coordinates^33^. Horizontal slices can reveal the soil layers, identify feature shapes, and investigate lateral relationships by separating certain depths. A circular well, a building foundation, or ventilation ports are examples of features whose shapes can be seen with the use of depth slices. The slices are often the best way to convey findings to the public and significantly expand the amount of interpretive information. The size, shape, and location of cultural relics buried at different depths can be ascertained with the help of three-dimensional analysis^22^.

## Data acquisition

### Site description

The surveyed archaeological site is located in the Quarn Ginah hillock. This hillock is a 204 m high, double-peaked black anticline hill surrounded by dunes (Fig. 1). It is a rugged sandstone hill that stands alone on the depression floor, visible from most locations, and serves as an excellent landmark. Archaeological and associated constructions have yielded a wide range of discoveries. The archaeological feedback about this site is very scarce, with no references. Even what is known about this site is not conclusive or reliable. The site has sustained significant damage from fire and destruction caused by the invaders (probably the Blemmyes in the fifth century AD)^34^.

### Field survey

**Fig. 5:**
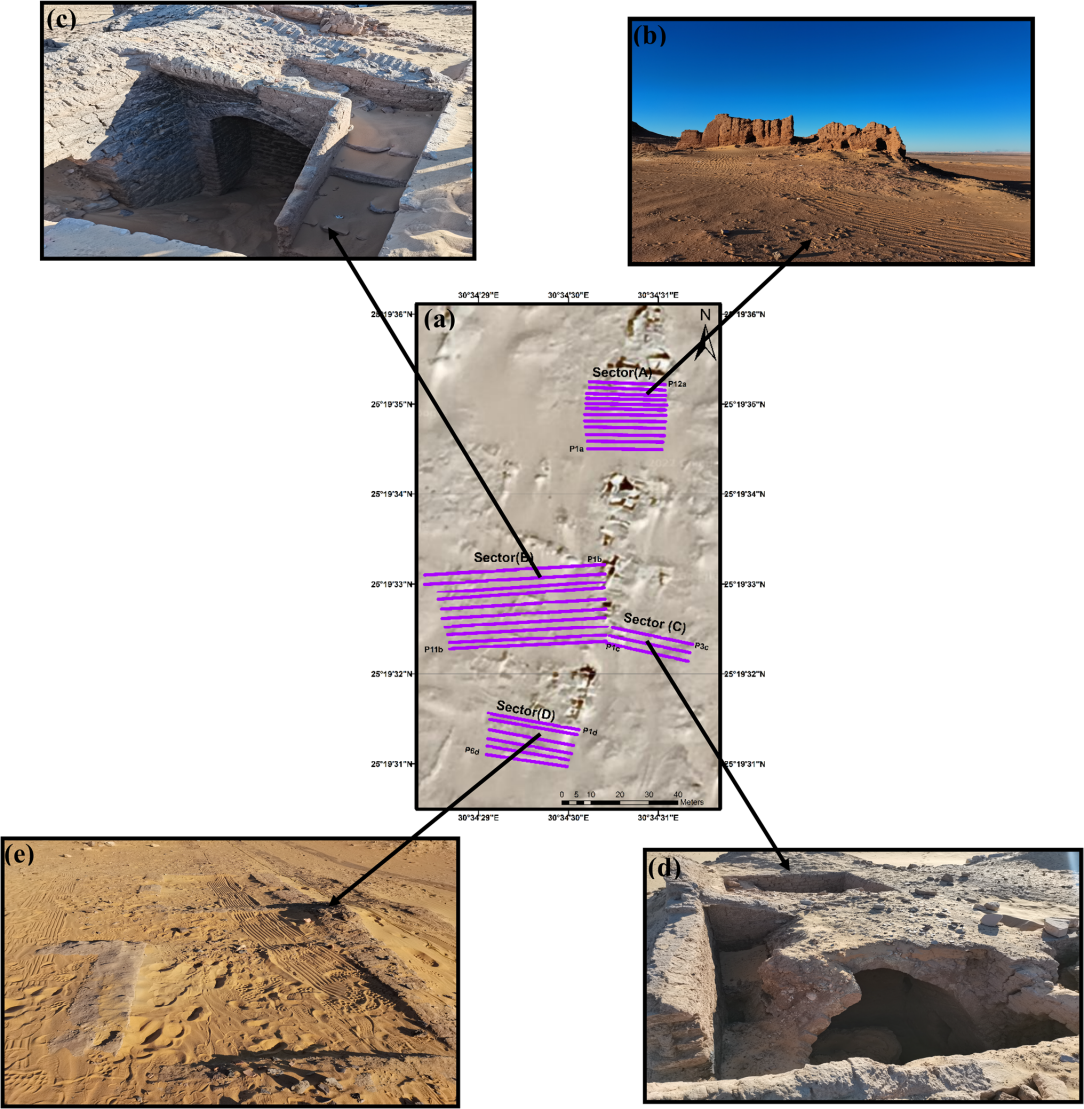
A map showing the layout of the measured GPR profiles with their grids at Ginah (**a**), a standing wall in sector A (**b**), extensive damage showing the broken chamber with a vault and downstairs in sector B (**c**), three broken chambers covered partially by sand with vaults in sector C d), and surface ruins of walls, chambers, or fences in sector D e).

A previously excavated site is near the 120 × 120 m field survey, and the GPR surveys were conducted to increase the subsurface information surrounding the already excavated site. The site was divided into four sectors (A, B, C, and D) to explore near-surface and buried archaeological findings in each one. Their locations were chosen close to existing archaeological ruins and between (Table 2 and Fig. 5). To the north of the examined area is Sector A, which is distinguished by ruins and rubble walls (Fig. 5b). Sector B represents unearthed broken sites lying west of the study area (Fig. 5c). Only one of its four fractured rooms, which are partially obscured by eolian sands and have vaults, is visible from the outside. Three of these chambers were built from mud bricks, and the fourth was built from sandstone, as observed in the ruins on the surface. Sector C lies east of Sector B (Fig. 5d). Sector D represents an unearthed and broken site lying south of the study area (Fig. 5e). It contains ruins of walls, chambers, or fences at the surface having unknown dimensions.

**Table 2:** Description of GPR profiles measured in different sectors in the surveyed area.

Surveyed site	Dimensions (M)	Profiles	
		No	Length (m)
Sector A	25 × 28	12	60
Sector B	28 × 63	10	25
Sector C	7 × 28	3	25
Sector D	16 × 32	6	28

**Fig. 6:**
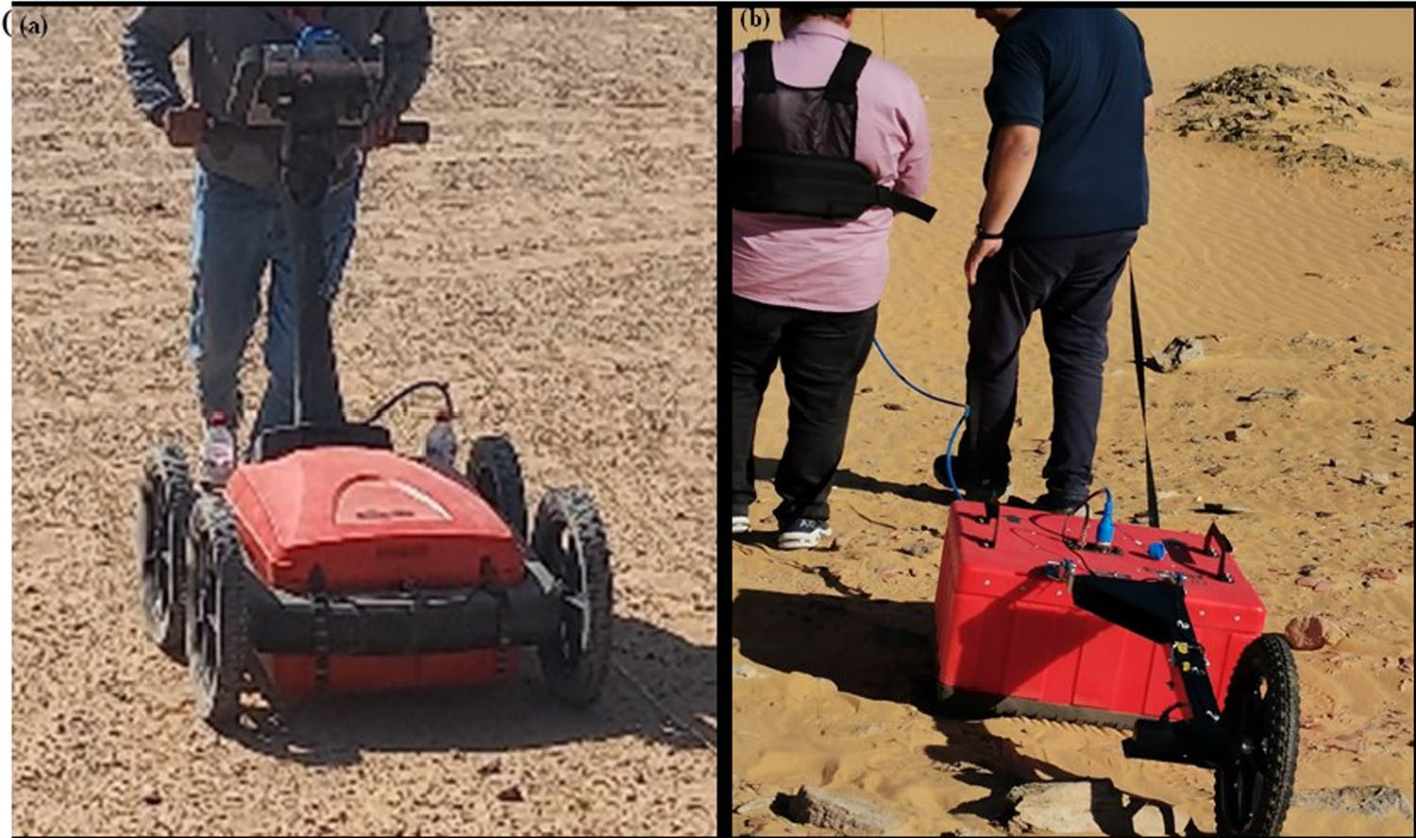
Photographs showing GPR field surveys using; 200 MHz (**a**) and 400 MHz (**b**) antenna.

Field measurements were performed using an antenna with nominal center frequencies of 200 and 400 MHz, the SIR System-4000, and the SIR-10b (control unit)^33^. The instrument works in monostatic mode with a constant antenna separation of 0.2 m. The corners of the survey sites were located using a GPS receiver (Maxor, USA) built into the RADAN 7 programmer^35^. All the data were recorded in ‘zigzag’ mode, along the east–west direction, with a spacing of 0.5 m. An odometer wheel measures the in-line coordinates, triggering the device every 0.05 m. The stack of eight, a temporal sampling interval of 0.2 ns, and a time window of 80 ns were used to record all of the GPR data. Because the goal is 3D imaging, distance-based data were collected at 200 MHz with a survey wheel (autoprofiling technique) (Fig. 6a). A reconnaissance GPR survey was carried out on the area for sector A using the autoprofiling technique (400 MHz antenna) (Fig. 6b).

### Processing

The field measurements were carried out as reflection profile, 2D processed radargram, depth slices, and 3D subvolume. Depth slices are the basis for displaying GPR data. This could be enhanced by using 3D visualization^21^. The final aim of GPR data preparation is to deliver and clarify an image that can be deciphered to distinguish archaeologically buried targets. The single 2D profiles are interpreted to produce a 3D image. For the 200-MHz antenna, a high-pass filter at 50 MHz and a low-pass filter at 600 MHz are applied. For the 400-MHz antenna data, a high-pass filter at 100 MHz and a low-pass filter at 800 MHz are used. By pressing the mark button at the anticipated depth of the archaeological remains (at 0.01 m resolution), the speed of the measurements was recorded. A 7–59 dB display gain was used to enhance the reflections and strengthen the weak signals.

**Fig. 7:**
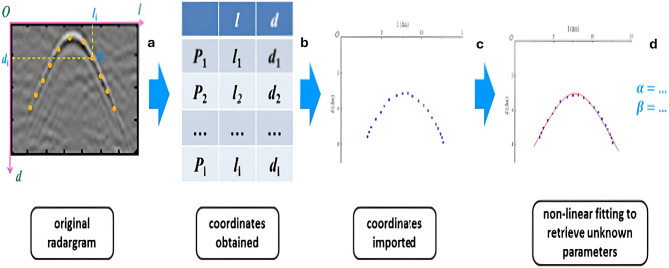
Stages of GPR radargram processing; curve fitting extracted along the hyperbolic signals in yellow dots (**a**), recorded coordinate pairs (**b**), coordinate data and equations that were imported into the software program (**c**), and completed curve fitting with figured-out relevant parameters (**d**).

The RADAN 7 program was used to enhance the original data’s quality and facilitate better interpretation. The main processing steps can be summarized as follows: (1) time-zero correction; (2) running an average filter with a length of 4 ns to filter the DC component; (3) AGC with a window length of 61 ns; (4) subtracting the mean trace to filter out the continuous flat reflections; (5) bandpass filter: 100/200–300/400 MHz; (6) constant gain function, wherever necessary; and (7) migration eliminates the hyperbola’s errant tails and precisely fixes the position of the target. Geometrical modeling of diffractions is required to estimate the EM wave velocity to determine the depth of archaeological remnants. GPR radargrams should be processed in the following steps: curve fitting extracted along the hyperbolic signals in yellow dots (Fig. 7a), recorded coordinate pairs (Fig. 7b), coordinate data, and equations that are imported into a software program (Fig. 7c), and complete curve fitting with calculated relevant parameters (Fig. 7d).

## Interpretation and results

The unique characteristics of reflection patterns, amplitude, continuity, and configuration are essential for identifying archeological features. The geometrical facies produced from various buried targets along the profiles under study should be integrated with all of these implications. These reflections originated from multiple sources: facies 1 to 6 (culture sediment), facies 7 (vaulted ceilings), facies 8 (walls), facies 9 (towers), facies 10 (floors), facies 11(staircases), facies 12 (rubble walls), facies 13 (rubble materials), facies 14 (cellars or wells), facies 15 (rubble chambers), facies 16 (courtyards), facies 17 (rubble silt and sand), and facies 18 (rubble walls). Additionally, by applying migration to the hyperbola, the corresponding culture layers and expected archaeological structures are identified (Table 3).

**Table 3:** Interpreted radar facies, elements, modelling parameters of culture sediments, and archaeological features from processed radargrams in the studied area.

Radar facies	Unit	Reflection			Geometry of facies unit	(ε)	V (m/ns)	Depth (m)	Width (m)	Building material	Expected layers/Archaeological features
		Amplitude	Continuity	Configuration							
1	–	High	High	Parallel stratified	Planar	2.26	0.01–0.29	0.0–1.5	–	–	Eolian sand
2	–	Low	High	Coherent parallel	Planar	24	0.058	1.5–2.5	–	–	Remains of fence or walls
3	–	High	Low	Parallel stratified	Planar	15.13	0.07	2.5–4	–	–	Silt
4	–	Low	Low	Longitudinal cross—bedding	Cross bedding	22	0.56	2.7	–	–	Individual fractures
5	–	High	Low	Chaotic	Cavern	5	13	0.1–0.07	–	–	Natural buried boulders
6	–	High	High	Parallel stratified	Lineal	–	–	1.5–2.5	–	–	strata
7	3	High	Low	Point source	Diagonal reflections	4.72	0.138	0.0,0.5,2.2	0.2	Mud break	Vaulted ceiling
8	3	High	High	Vertically stacked reflections	Lineal	7.14	0.112	2,4,6	0.8	Lime stone	Walls
9	2	High	High	Vertically stacked reflections	Lineal	8.79	0.101	4,8	1.2–2	Lime stone with sandstone	Tower
10	4	High	Semi to continuity	Flat to slightly	Large spatial	4.72	0.138	2,4,6,8	0.2 – 0.6	Mud break or/and sandstone	Floor
11	5	High	Low	Oval	Oval	–	–	7–8	0.7	Sandstone	Staircase
12	–	High	Continues	Chaotic	Mounded	–	–	0.0–8	10	Mud break	Rubble hearth
13	2	High	Low	Chaotic	Semi oval	15.13	0.077	4,7	0.2–1	Clay soil saturated	A chaotic mixture of rubble
14	1	High	High	Vertically stacked reflections	Lineal	4.72	0.138	4	4.2	Mud break with sandstone	Cellar/well location
15	3	No significant reflection			Rectangular	–	–	0.5	4–10	Mud break	Rubble chamber
16	1	No significant reflection			Rectangular	–	–	6	5	Filled with silt	Courtyard
17	1	Low to medium	Discontinues	Undulation	Undulation	12.51	0.085	0.8	Filled the vaulted chamber	Silt and sand saturated	Rubble Silt and sand saturated
18	1	High	Semi to continuity	Vertically stacked reflections	Distorted lineal	7.38	0.110	3.2	Distorted lineal	Mud break or/and sandstone	Rubble Walls or columns

V: EM velocity, ε: relative dielectrical permittivity.

### Sector A

**Fig. 8:**
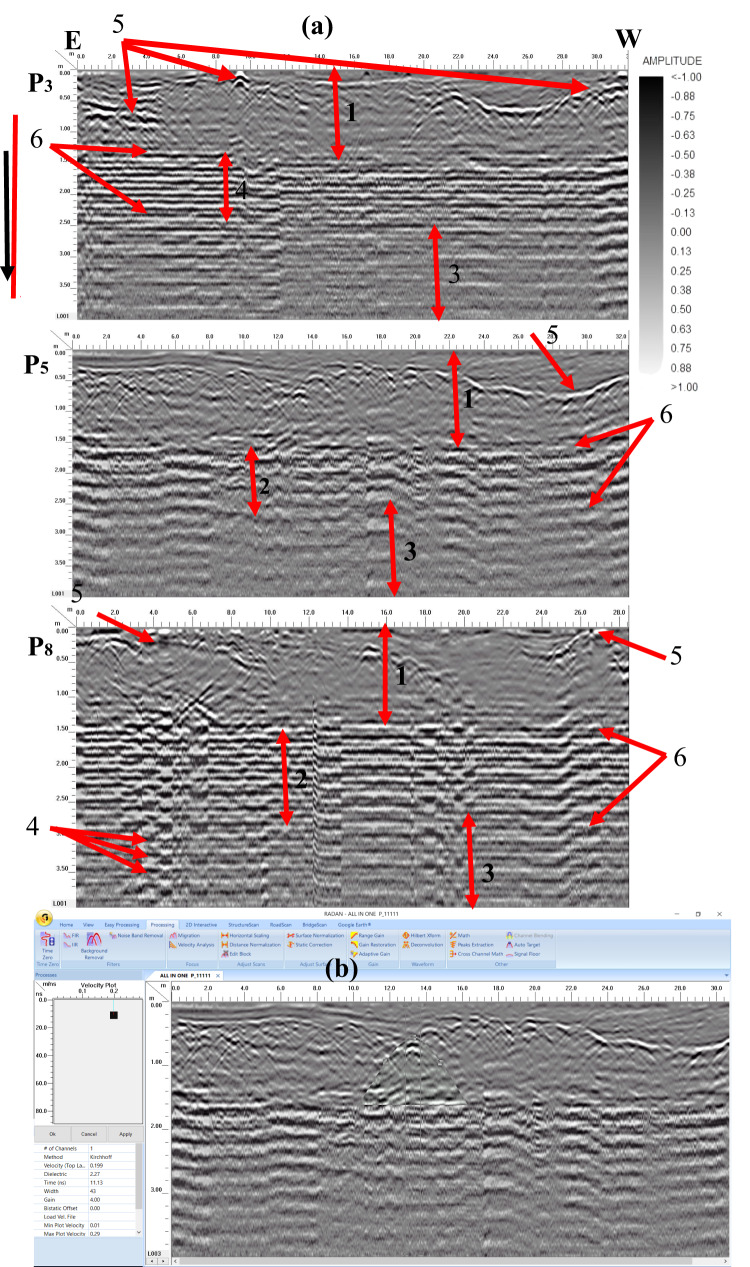
Examples of 2D-processed GPR radargrams (**a**) and modelling parameters (**b**) for sector A using a 400 MHz antenna.

The following is an interpretation and identification of six georadar facies with clear stratigraphic discontinuities (Fig. 8a) and the cultural layers that correlate to them (Fig. 8b):

- Georadar facies (facies 1) are characterized by high reflection amplitudes, high-continuity reflections, parallel stratified reflection configurations, and planar geometric facies. It may represent eolian sand from the surface to 1.5–2 m.
- These facies (facies 2) are distinguished by low reflection amplitude, high-continuity reflections, a coherent parallel reflection pattern, and planar geometric facies. The facies may indicate the remains of fences or walls below facies one with sharp contact to a depth of approximately 2.5–3 m. This suggestion is based on the affinity of exposed walls in this studied sector (Fig. 5b).
- Georadar facies (facies 3) display weak reflection amplitudes, reflections with limited continuity, parallel stratified reflection patterns, and planar geometric facies. It is below the facies 2 and may reflect silty sediments to a depth of 4 m (maximum penetration).
- Radar facies (facies 4) is defined by weak reflection amplitudes, discontinuous reflections, reflections indicative of longitudinal cross-bedding, and a sheet-like geometry. It may represent individual fractures crossing the facies 2 to a depth of 3–4 m (maximum penetration).
- Radar facies (facies 5) are characterized by high reflection amplitudes, low-continuity reflections, chaotic reflection configurations, and cavern geometry. It is disseminated in facies 1 and may reflect naturally buried boulders or gravels.
- Radar facies (facies 6) are characterized by high reflection amplitude, high-continuity reflection, a parallel stratified reflection configuration, and lineal boundary. It may represent the boundary between layers.

### Sector B

**Fig. 9:**
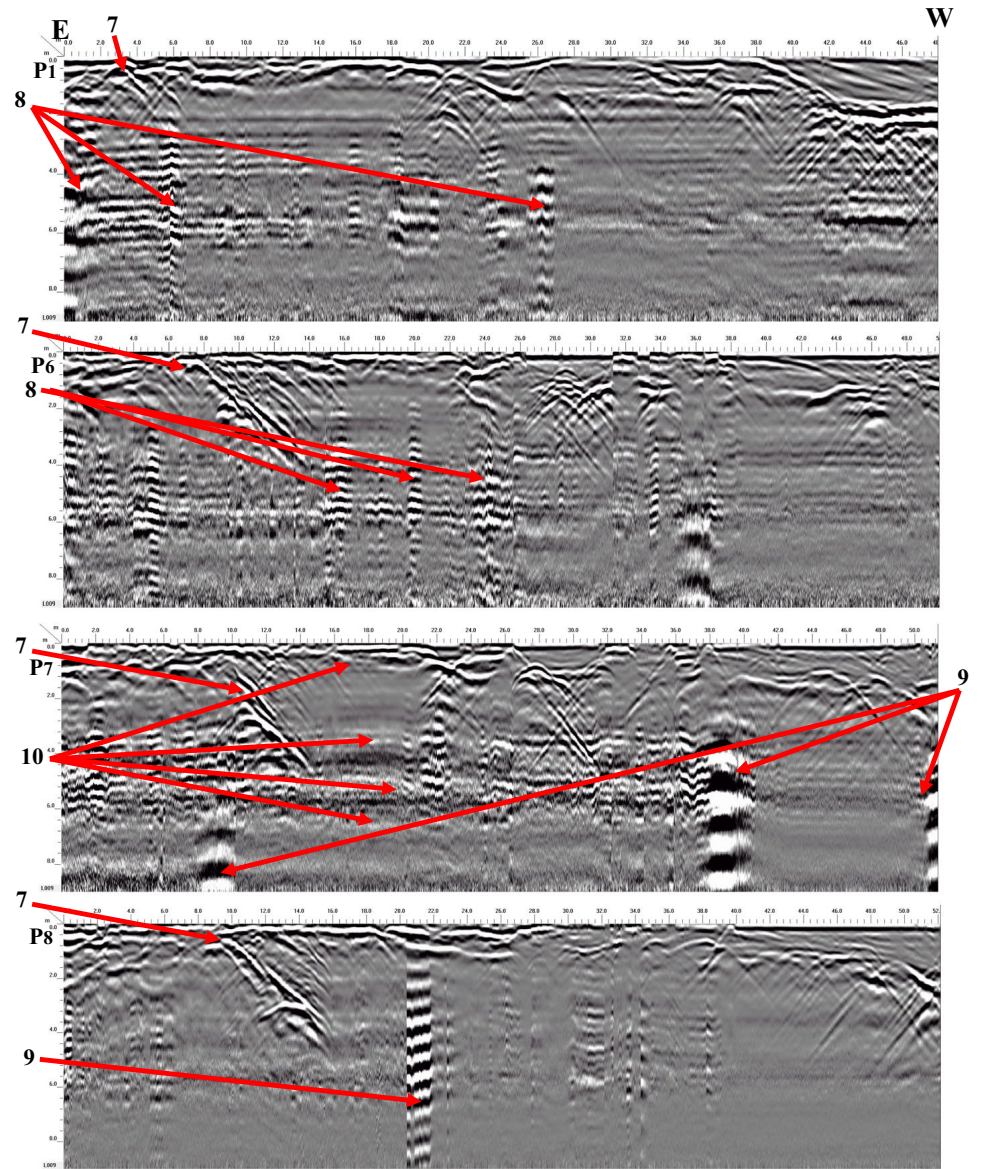
Examples of 2D-processed GPR radargrams for sector B using a 200 MHz antenna.

**Fig. 10:**
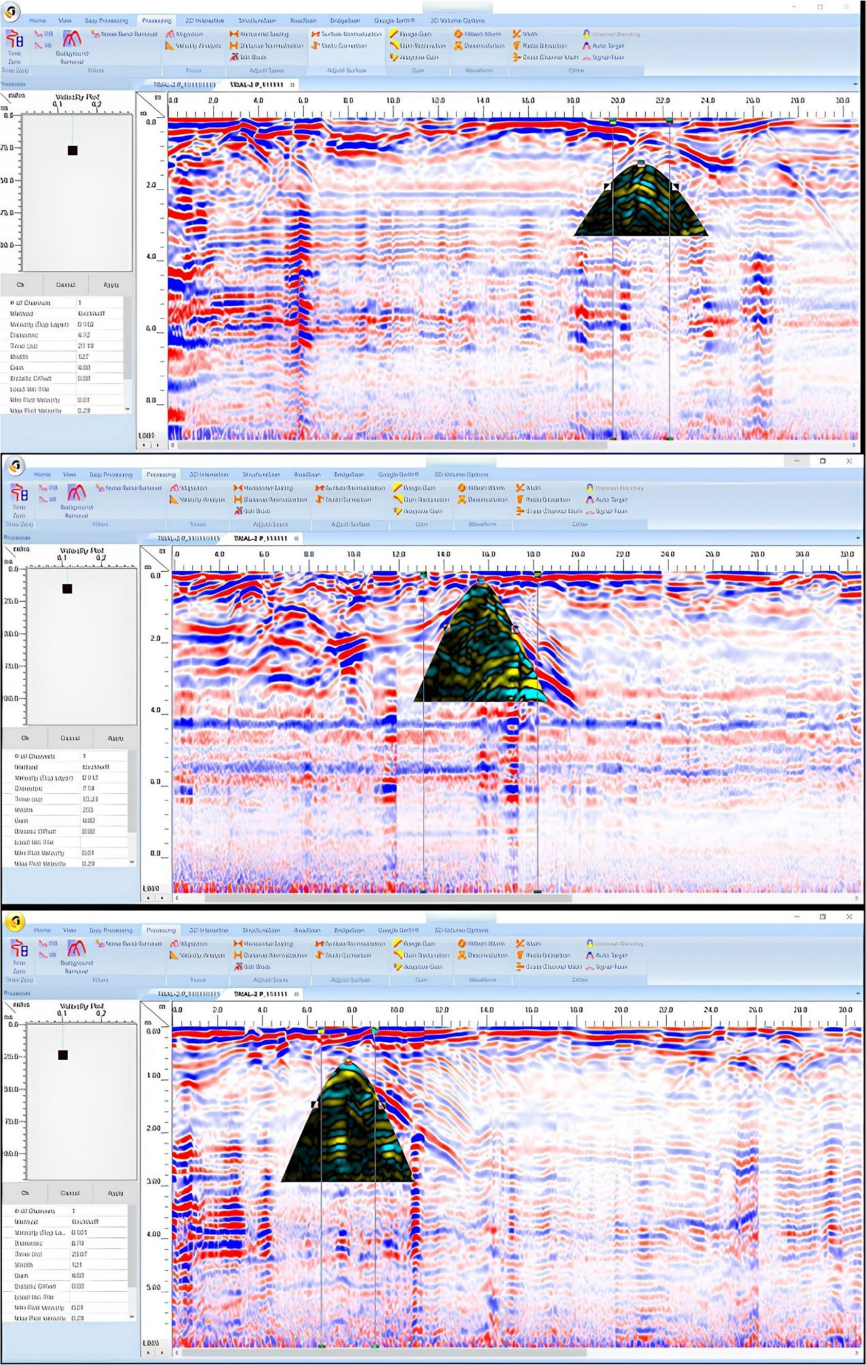
Modelling parameters attached to each buried feature for sector B.

Four georadar anomalies (facies 7–10) (Fig. 9) and their corresponding expected archaeological structures (Fig. 10) are interpreted and identified as follows:

- Vaulted ceilings (facies 7, mud bricks) produce half of the hyperbolas dipping towards one another from the upper edge of the burial feature (high amplitude, low continuity, point source configuration, and diagonal reflection). They appear at two levels (ground surface and 5 m).
- The walls create reflections (facies 8) that are stacked vertically, culminating in a hyperbola (characterized by high amplitude, high continuity, a vertically stacked arrangement, and linear geometry) that support the vault. These walls are present at three different heights: 2 m (constructed from mud brick), 4 m, and 6 m (made of limestone).
- Towers (facies 9) have high amplitude, high continuity, a vertically stacked configuration, and lineal geometry. They appear at depths of 4 m (limestone) and 8 m (sandstone).
- Floors or ceilings (facies 10) produce high reflection amplitudes, semi-to-continuity reflections, flat-to-slight configurations, and large spatial geometries. They appear at depths of 2 m (mud brick), 4 m (limestone), 6 m (limestone), and 8 m (sandstone).

**Fig. 11:**
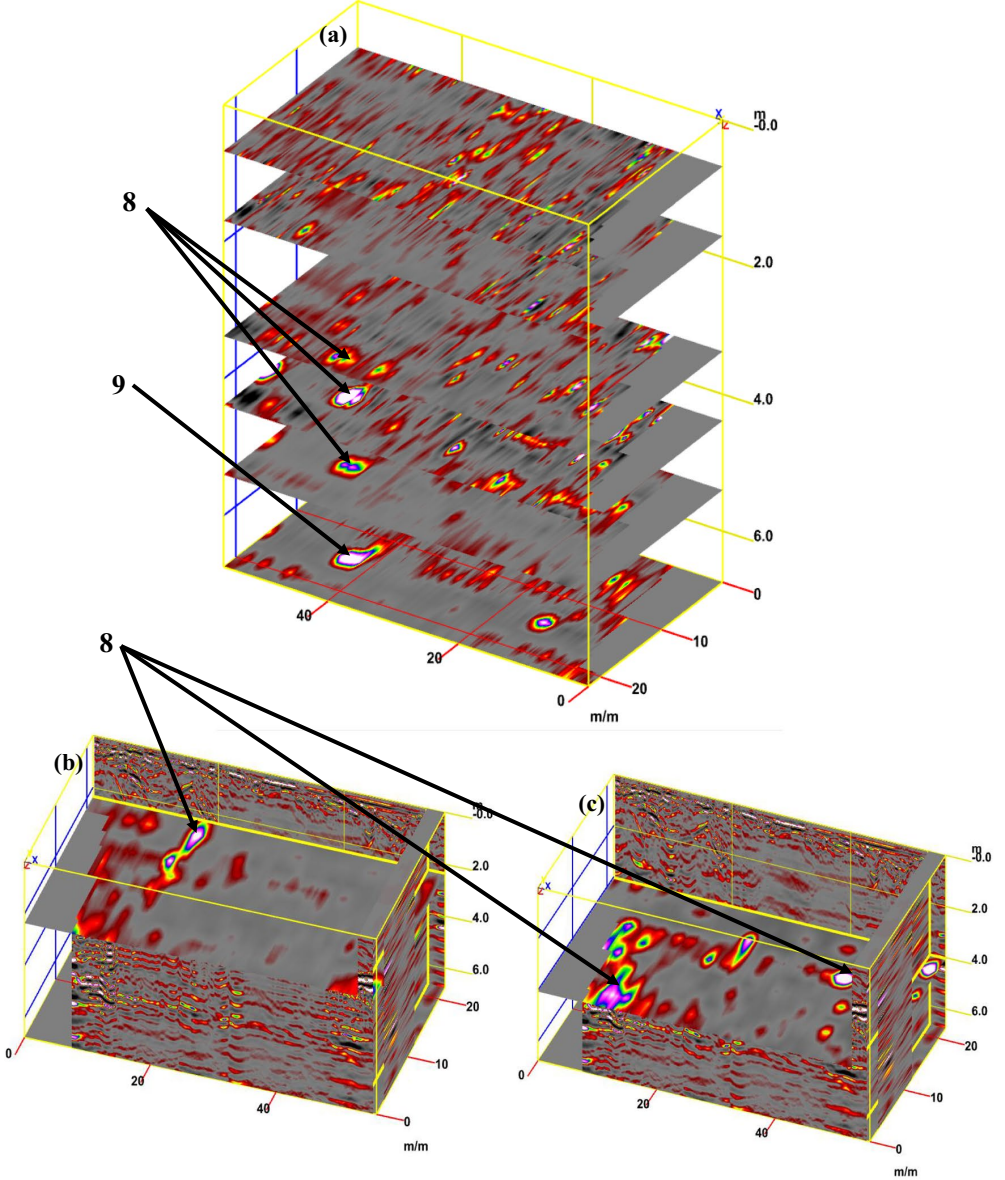
Creation of depth slices from multiple 2D reflection profiles of sector B; from surface to 8 m depth (**a**), the 3D GPR sub-volume shows large lateral and vertical variations: the energy attribute plotted on the 46 ns time slice highlights some possible lateral continuous structures not clearly detectable on the amplitude data analysis of vertical sections, the celling of the chamber at depth 2 m (**b**) and its floor at depth 4 m (**c**).

GPR depth slices obtained via RADAN 7 are applied to assess and interpret the field data further (Fig. 11a). A new ceiling model was created to confirm that the diagonal reflections were produced by the interface between the air and the ceiling (2 m depth), where they do not appear in all profiles, and to assess what the reflection result would be from a possible structural phase (Fig. 11b). To achieve a more dependable result, the new model was built using the same shape and dimensions as the previously discovered portion of the floor (4 m depth) (Fig. 11c). Figure 11 presents the new simulated ceiling and floor model, which includes two different construction phases, an area with high reflection content, a distorted area (air-filled cracks), bricks, and inhomogeneous mortars to produce micro reflections. The characteristics and building materials used for the ceiling and floor are shown in Table 3.

**Fig. 12:**
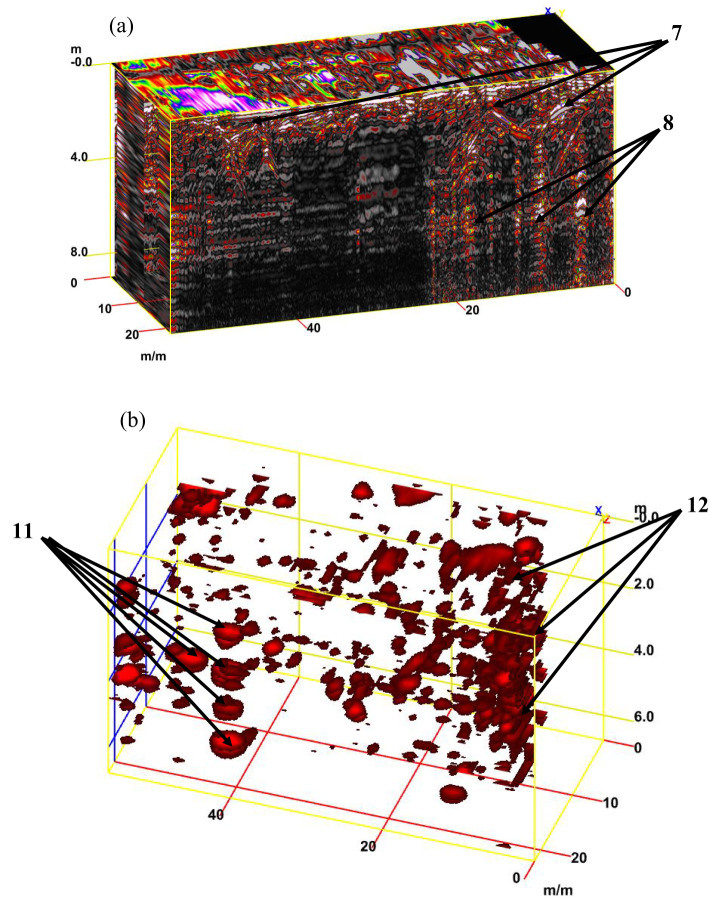
3D GPR data display of sector B showing the extension of the carrying layer of archaeological objects (**a**) and the corresponding 3D data volume showing only high-amplitude data points (**b**).

Figure 12a presents a synthetic 3D GPR subvolume cube deduced from the data display of sector B, showing lateral and vertical variations of; the vault ceiling (facies 7); and walls (facies 8). Figure 12b shows a strong horizontal reflection that can be seen in the radargram, which correlates to the different construction phases incorporating; the staircase (facies 11) and the rubble hearth (facies 12). Furthermore, multi-diagonal reflections originated from the lateral end of the rubble hearth. Finally, the distorted, cracked area is also identified as a small staircase with strong oval reflections.

### Sector C

**Fig. 13:**
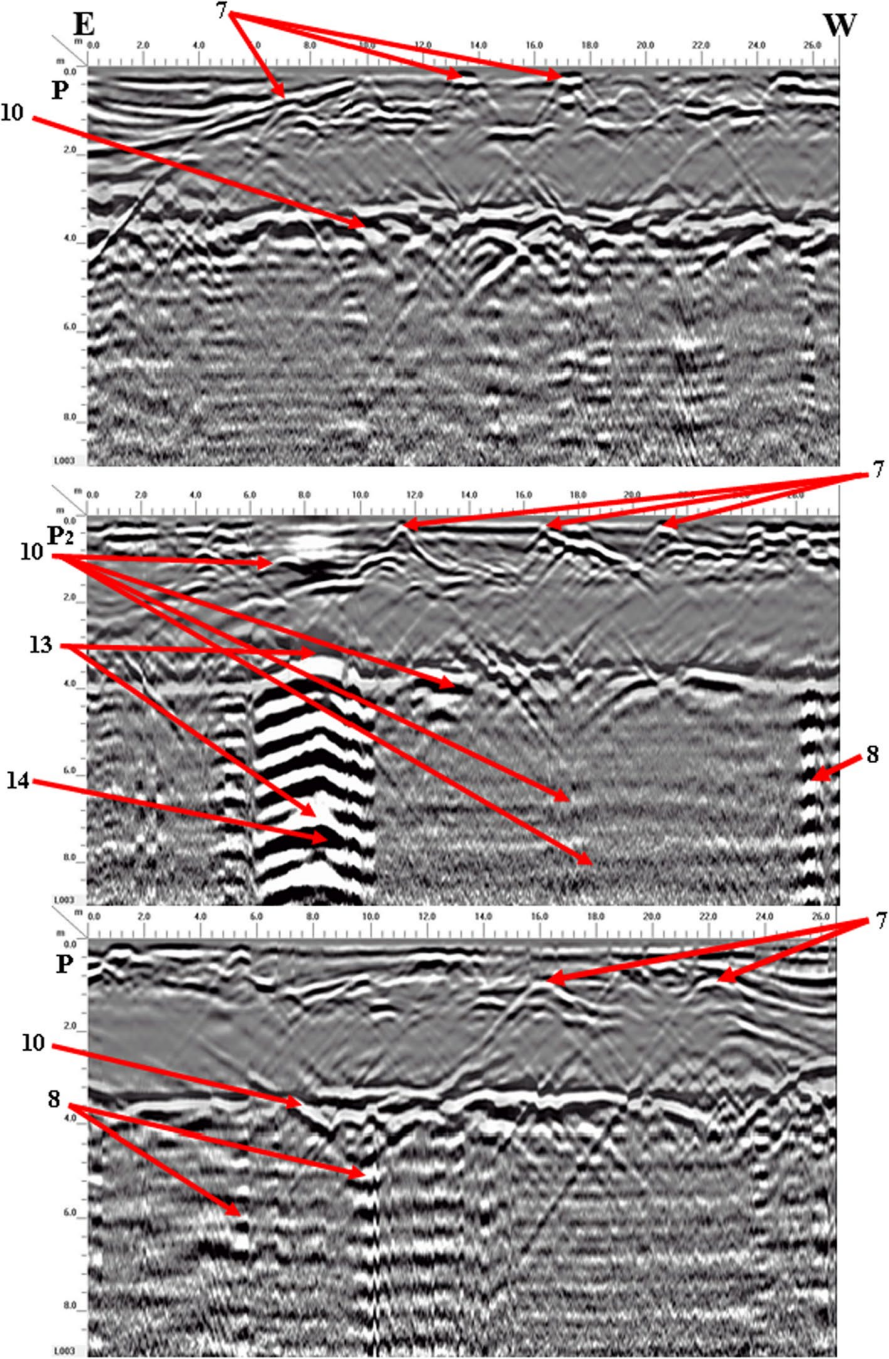
2D-processed GPR radargrams for sector C using a 200 MHz antenna.

**Fig. 14:**
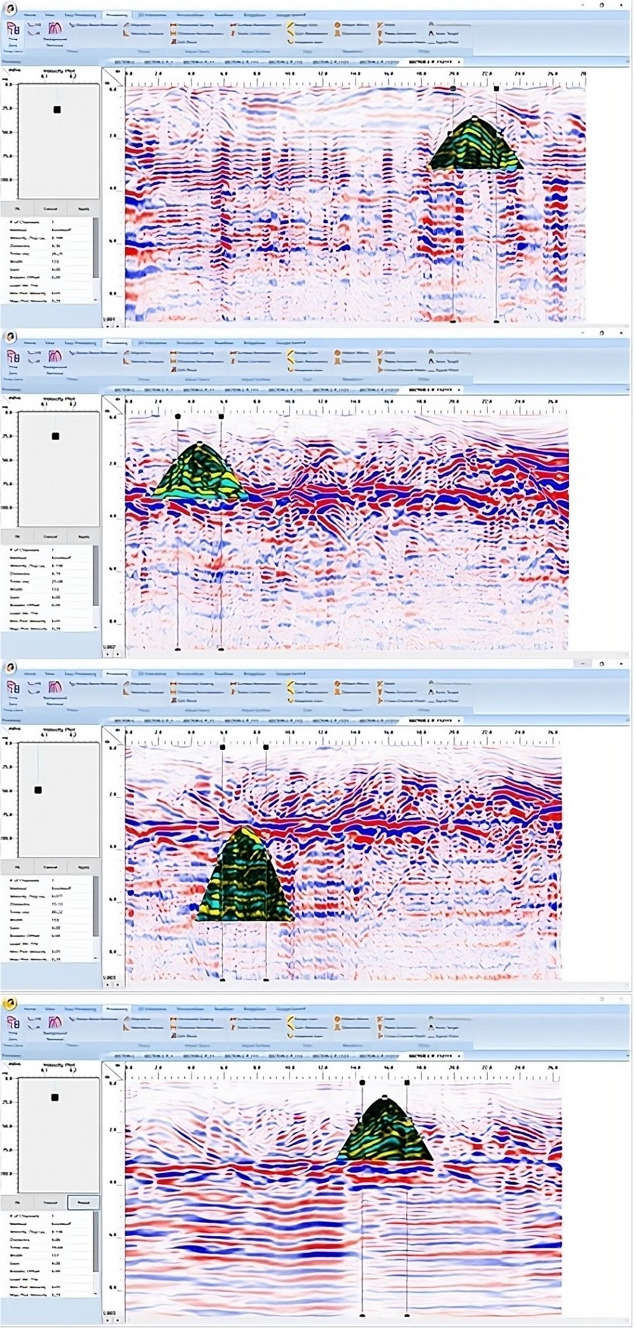
Modelling parameters attached to each buried feature for sector C**.**

Seven georadar anomalies (facies 7, 8, 10, 13, 14, 15, and 16) (Fig. 13) and their causative expected archaeological sources (Fig. 14) are determined and explained as follows:

- Facies 7, 8, and 10 are the same as those mentioned in sector B, but they appear faintly in the processed radargrams. Only facies 8 and 10 appear at different levels.
- A chaotic mixture of rubble (facies 13) creates strong, low-coherence reflection patterns and semi-oval geometric shapes, observable at two depths: 4 m, where compacted materials outline the framework of the cellar or well hole, and 7 m, within the cellar or well itself.
- The cellar or well (facies 14) displays strong amplitude, consistent reflections, a vertically aligned geometric shape, and a linear structure. It seems to be situated at a depth of 4 m and has a width of 2 m.

**Fig. 15:**
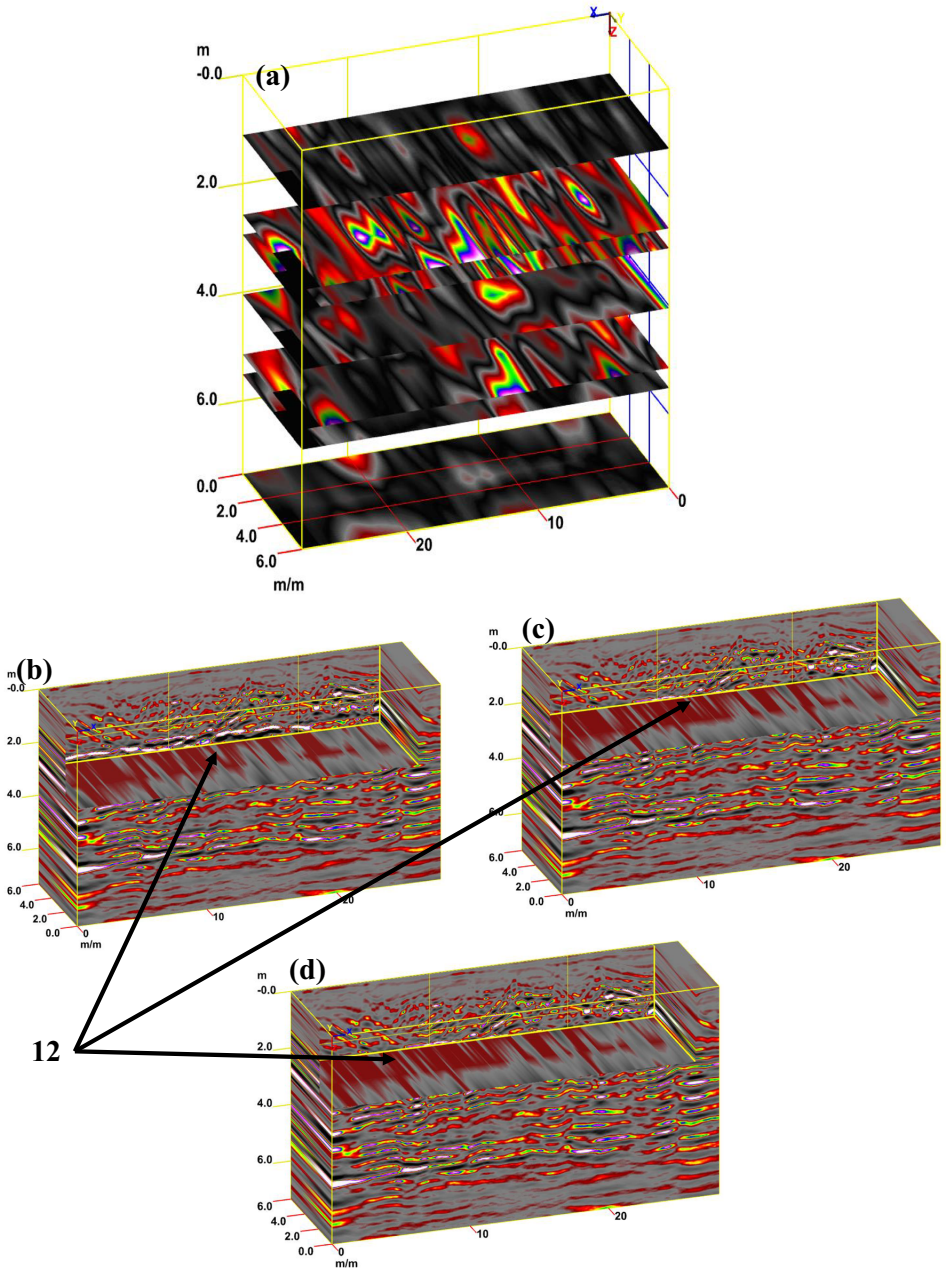
Creation of depth slices from multiple 2D reflection profiles of sector C; from surface to 8 m depth (**a**), 3D GPR sub-volume shows large lateral and vertical variations: the energy attribute plotted on the 46 ns time slice highlights some possible lateral continuous structures not clearly detectable on the amplitude data analysis of vertical sections, tracking the archaeological object in profile 1 at depth 2 m (**b**), in profile 2 at depth 2.5 m (**c**), and in profile 3 at depth 2.5 m.

**Fig. 16:**
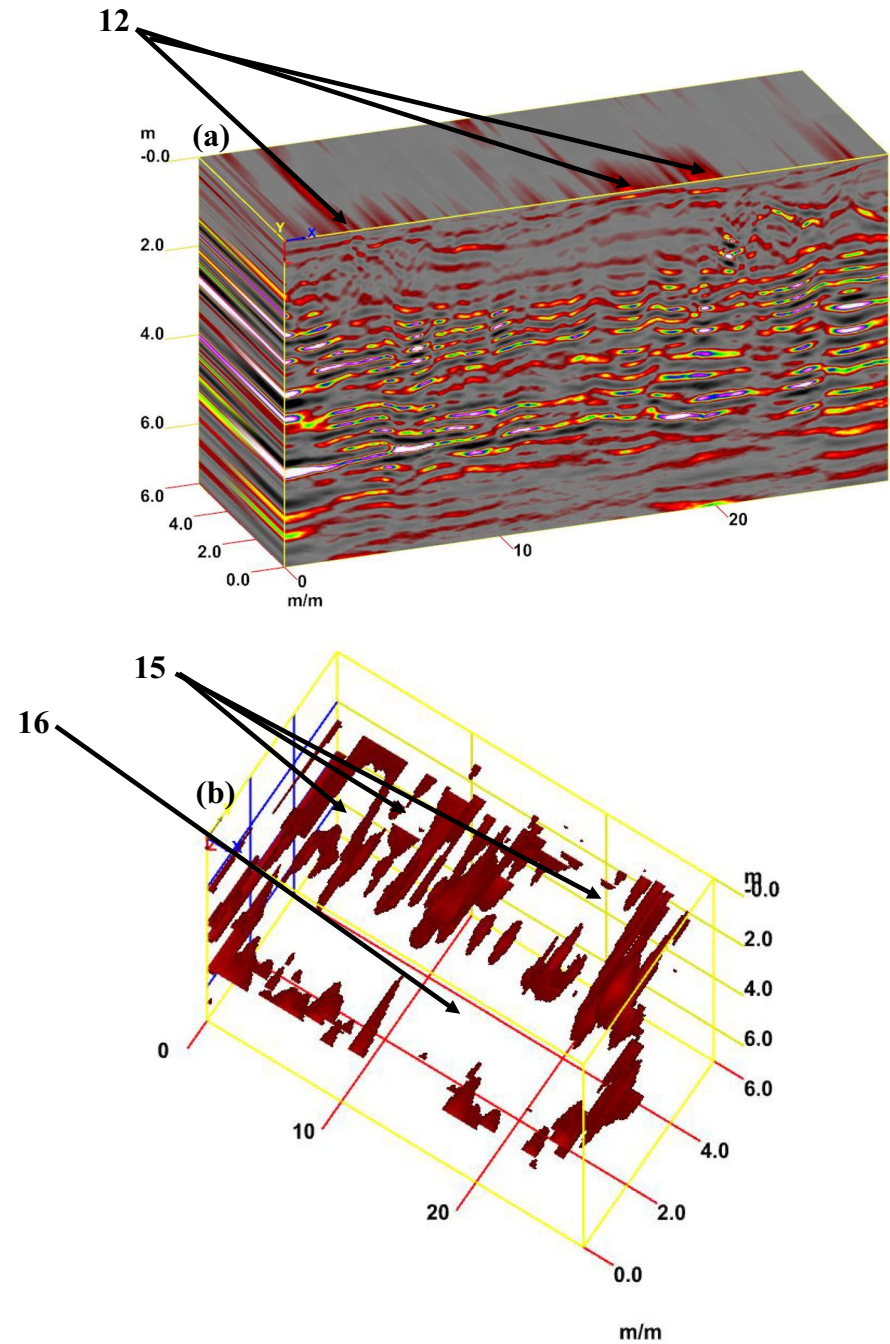
3D GPR data display of sector C showing the extension of the carrying layer of archaeological objects (**a**) and the corresponding 3D data volume showing only high-amplitude data points (**b**).

For tracking archaeological structures in sector C, depth slices from the surface to 8 m or more are created from multiple 2D reflection profiles (Fig. 15a). The inspection of these slices revealed the presence of rubble walls (facies 12) in profile 1 at a depth of 2 m (Fig. 15b), profile 2 at a depth of 2.5 m (Fig. 15c), and profile 3 at a depth of 2.5 m (Fig. 15d). Figure 16a presents a synthetic 3D GPR subvolume cube deduced from the data display of sector C, showing lateral and vertical variations in the rubble wall (facies 12) (Fig. 16a). Figure 16b shows a strong horizontal reflection, which may be due to different phases of construction; rubble chambers (facies 15) and rectangular courtyards (facies 16). The distorted, cracked areas identify three broken chambers at depths of 2 m and 6 m in length. Two of these chambers are 4 m wide (one has three completed walls, but the second has some remaining walls), and the third is approximately 10 m wide and has some remaining walls. The courtyard may appear in the 3D data volume with no significant reflection (at 5 m depth, 5 m width, and 22 m length), presumably depending upon the construction of the footpath compared with the surrounding materials. This courtyard may be used as a space for rituals.

### Sector D

**Fig. 17:**
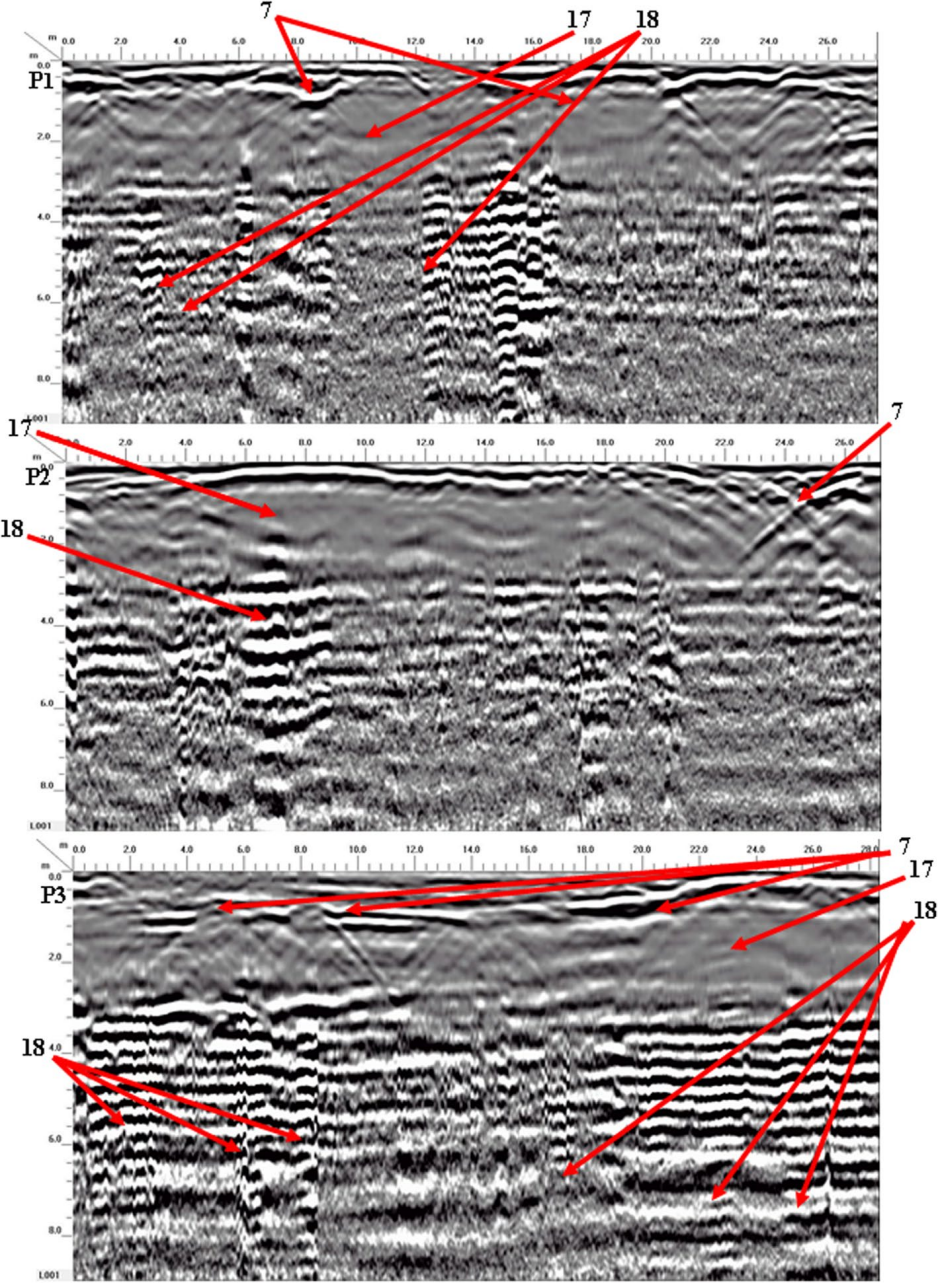
Examples of 2D-processed GPR radargrams for sector D using a 200 MHz antenna.

**Fig. 18:**
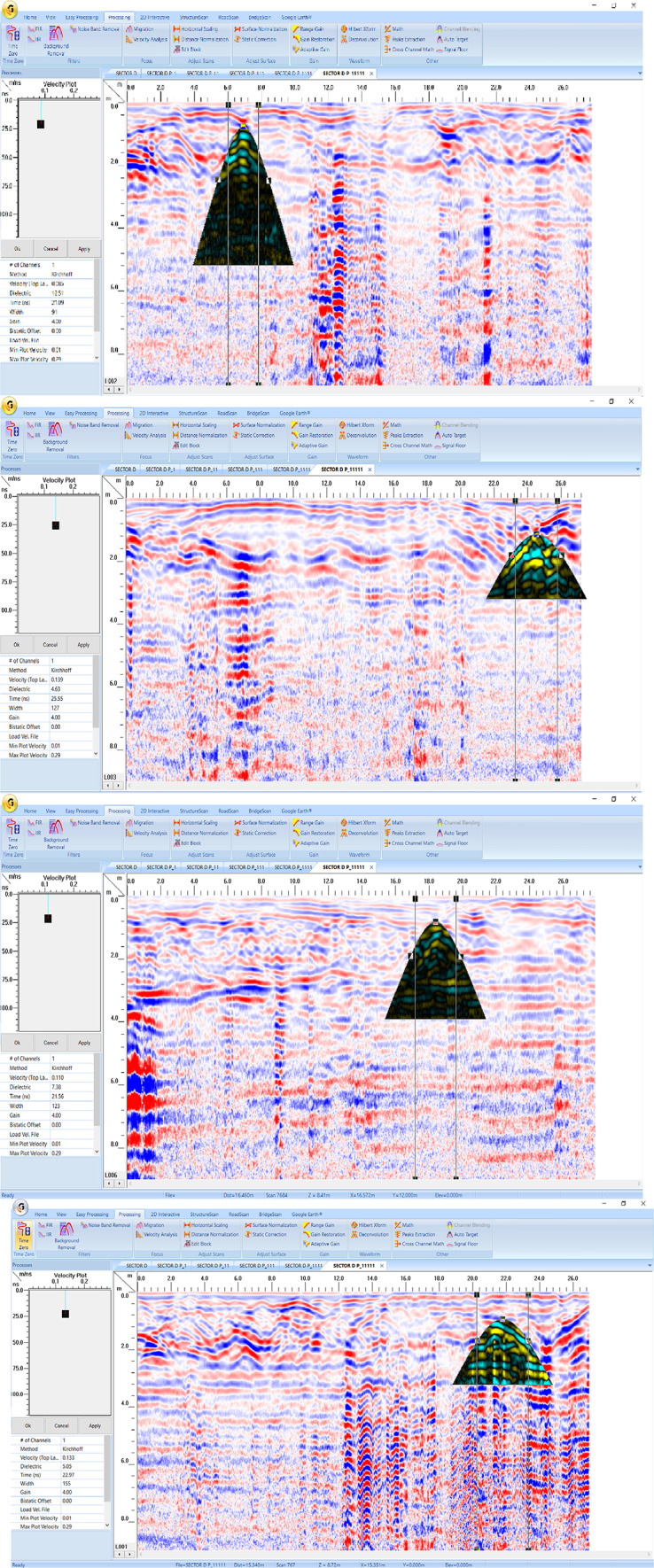
Modeling parameters attached to each buried features for sector D**.**

Figure 17 shows the presence of three georadar facies (7, 17, and 18), and their expected archaeological structures are shown in Fig. 18:

- Rubble and moister sediments (facies 17, sand, silt, and clay) generate varying amplitudes of reflections, ranging from low to high, with discontinuous, undulating reflection patterns, and distinct geometrical forms. It appears that the space between the air and the arched ceiling is filled with a single layer (8 m).
- Rubble walls or columns (facies 18) produce high-amplitude, semi-to-continuous reflections, vertically stacked reflections, and distorted lineal geometric shapes. They appear at 3.2 m (mud brick or sandstone).

**Fig. 19:**
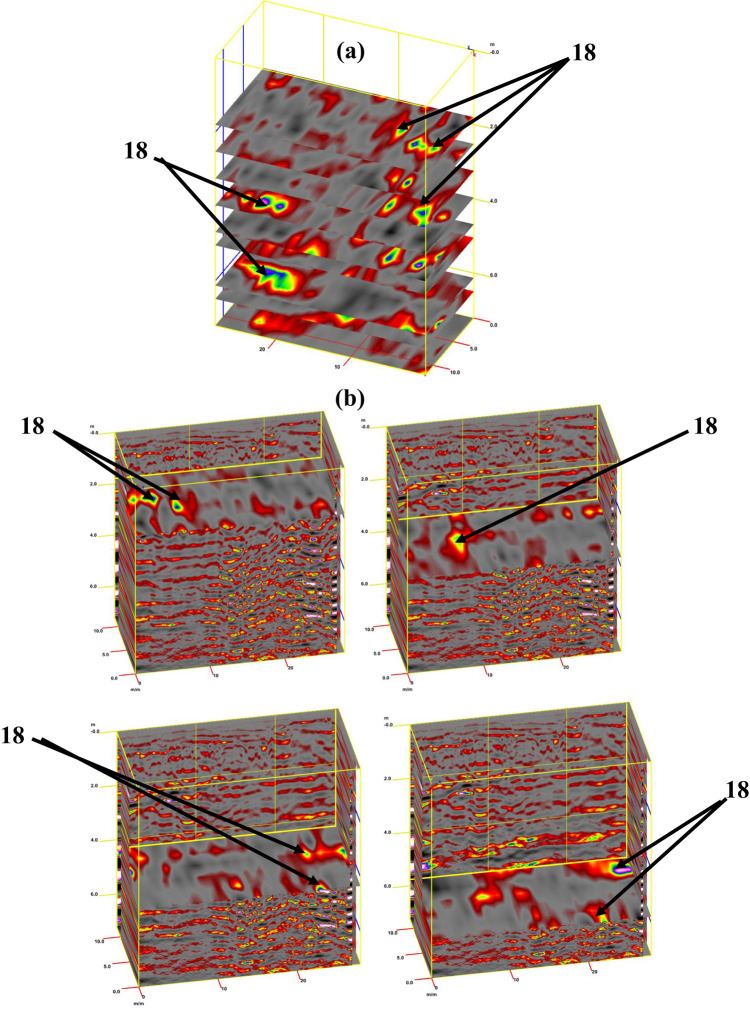
Creation of depth slices from multiple 2D reflection profiles of sector C; from surface to 8 m depth (**a**), 3D GPR sub-volume shows large lateral and vertical variations: the energy attribute plotted on the 46 ns time slice highlights some possible lateral continuous structures not detectable on the amplitude data analysis of vertical sections and display the extension of the carrying layer of archaeological objects (**b**).

**Fig. 20:**
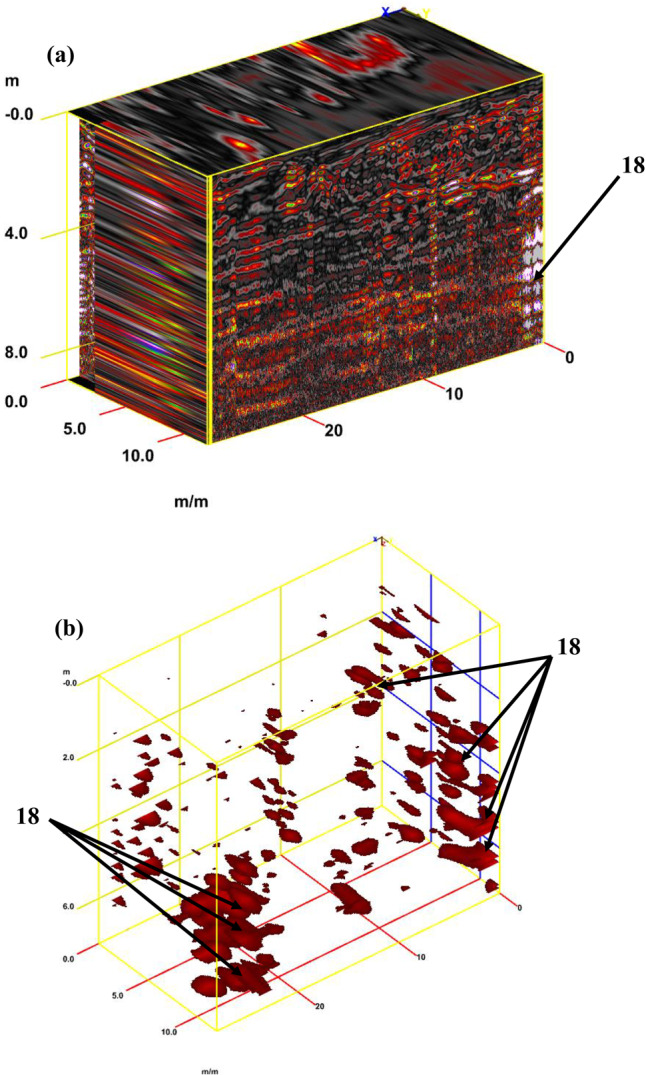
3D GPR data display of sector D showing the extension of the carrying layer of archaeological objects (**a**) and the corresponding 3D data volume showing only high-amplitude data points (**b**).

For tracking archaeological structures in sector D, 2D depth slices from the surface to 8 m or more and 3D depth slice cubs are created from multiple 2D reflection profiles (Fig. 19a and b). Inspection of these figures reveals the presence of rubble walls or columns at different depths. Figure 20a presents a synthetic 3D GPR subvolume cube deduced from the data display of sector D, which shows lateral and vertical variations in the rubble walls or columns (facies 18) (Fig. 20a). Figure 20b shows a strong horizontal reflection, which may be due to rubble walls or columns at different depths. The distorted, cracked areas identify many rubble walls or columns.

## Discussion

A GPR field survey on the Ginah area was conducted to obtain digital datasets in 2D (processed radargrams) and 3D (depth slices and subvolumes) formats. These processes are essential for excavation studies to determine soil types, burial features, variable states of preservation, and the possibility of having multi-story buildings in the investigated site. Additionally, it is beneficial to obtain the highest resolution data with the highest percent coverage for each specific target at different levels (from the ground surface to > 8 m).

The results of the GPR data on the area clearly distinguished between the georadar facies arising from the cultural layers (eolian sediments) and the buried archaeological features at a depth of 4 m. This geological medium provides sensitive vertical and horizontal resolution with satisfactory depth penetration. The comparison between the profiles measured in sectors B, C, and D with the reconnaissance profile in sector A allows us to determine the best antenna for the field survey and the vertical and horizontal positions of the buried archaeological remnants. Therefore, the shielded 200 MHz antenna was used to reduce the artificial effects of these sediments in the surveyed area.

Using GPR data from the associated ruins, a 3D distribution of the archaeological remnants was constructed to ascertain their dimensions, forms, depths, extensions, and building materials in a grid-like manner. The structures detected using GPR profiles provided a clearer understanding of the architectural layout of this site, enabling archaeologists to speed up their excavation process.

The vaulted ceiling is present in all sectors at two depths (from the ground up to 5 m). Since mud brick was a common building material throughout the Coptic era, it is conceivable that it was constructed during that time. The tower at a depth of 4 m may have been constructed during the Roman period; it is also made up of mud brick, but the underlying tower (8 m deep) may have been built during the Pharaonic period and made up of sandstone. The floor, which lies at a depth of 8 m, was constructed, including the top of the tower underlying it. This is evident from the radar reflection anomalies and the differences in building material between the tower and floor. The presence of a floor that is detected at different depths (2, 4, 6, and 8 m) may indicate different periods.

A chaotic mixture of rubble inside the cellar, well, walls, and columns appears only in sectors C and D, respectively. These archaeological features probably collapsed due to many factors, such as the weathering process and vandalism (mainly by bandits, Bedouins, Nobatae, and Blemmyes). These remarks were deduced from archaeological history, field observations, GPR data interpretation, and ruins around Quarn Ginah.

Ginah Hillock appears to have been created by several destroyed and rebuilt structures. Vertical superimposition of several structures was common in different periods at this site. This is associated with multiple periods of building construction, renovation, destruction, and redevelopment which can produce a complex series of archaeological layers that contain remains from different archaeological periods. Buried archaeological ruins are likely superimposed on several irregular layers and filled with thin mixed eolian sediment. Different materials, such as mud bricks, limestone, and sandstone with different architectural styles, are buried at different depths in the study area (> 8 m). The shape and function of the fortress at the Ginah archaeological site probably changed with different periods because it has different architectural styles at different levels (ground surface to > 8 m). The historical evidence suggests that different construction phases based on observed radar reflections may correspond to the interface between the four construction periods. These four broad stages of archaeological architecture development can be discerned within each culture: Coptic, Ptolemaic, Roman, and Pharaonic.

## Conclusion

- This study shows that GPR processing can improve the imaging and characterization of archaeological features under complex subsurface conditions.
- Deeper archaeological information can be obtained through GPR critical analysis and by overcoming the masking effect due to the shallower diffuse scattering zones (eolian sediments).
- A comprehensive near-surface geophysical survey on Ginah Hillock revealed that it was built over an earlier structure, whose architectural plan is certainly consistent with forts without the benefit of excavation, it is impossible to say what it was or when it was built.
- There are many different buried architectural features with their associated anomalies. Their fatigued descriptions of each one are probably beyond any researcher’s capabilities.
- Irregularly superimposed and interconnected archaeological structures at this site, are challenging to describe, follow, and map.
- Owing to insufficient information from preliminary excavations and the variable depth of the top of the buried remains, it is still difficult to correlate all the structures and establish a general subsurface archaeological map for different cultures at different levels.
- The authors look forward to future studies to fill the gaps between different ancient periods and remove the mystery to shed further light on these complex strategic operations that focused on the Kharga Oasis throughout different ancient periods.

## Data Availability

The datasets used and/or analyzed during the current study are available from the corresponding author on reasonable request.
